# Knowledge, Attitude and Practice (KAP) of Malay Elderly on Salt Intake and Its Relationship With Blood Pressure

**DOI:** 10.3389/fpubh.2020.559071

**Published:** 2021-02-04

**Authors:** Hasnah Haron, NurAisyah Farhana Kamal, Hanis Mastura Yahya, Suzana Shahar

**Affiliations:** ^1^Nutritional Science Programme, Faculty of Health Sciences, Universiti Kebangsaan Malaysia, Kuala Lumpur, Malaysia; ^2^Dietetics Programme, Faculty of Health Sciences, Universiti Kebangsaan Malaysia, Kuala Lumpur, Malaysia

**Keywords:** elderly, salt, hypertension, awareness, blood pressure

## Abstract

Hypertension is a worldwide problem and a major global health burden with high salt intake as one of the factors often related to it. Public exposure to a high salt diet has contributed to the increase in prevalence of hypertension among the Malaysian population. Improving the knowledge, attitudes and practices (KAP) related to salt intake is a key component of effective blood pressure control. Therefore, this study was aimed to determine the association of KAP of healthy salt intake toward blood pressure among the elderly residing in a semi urban area of Klang Valley Malaysia. A cross-sectional study using convenience sampling was conducted among Malay elderly, aged 60–81 years old residing in Bandar Baru Bangi. Subjects were required to answer the questionnaire via face to face interview regarding KAP of Salt Intake, together with sociodemographic and health profiles. Anthropometry parameters and blood pressure were measured. A total of 94 elderly [57.5% women with mean Body Mass Index (BMI) of 26. 46 ± 4.16, 42.5% men with mean BMI of 27.31 ± 5.19] have participated in the study. Results indicated that the overall KAP score was average (57.4%). However, majority showed a positive attitude toward reducing the salt intake. BMI was found to have significant correlation with blood pressure (*r* = 0.278, *p* < 0.05). Higher education level was significantly (*p* < 0.05) associated with good knowledge scores while younger subjects have a positive attitude toward healthy intake of salt (*p* < 0.05). Subjects with higher knowledge scores were also observed to have the more controlled blood pressure compared to those who scored less. Attitude and practices score showed no significance difference (*p* > 0.05) with blood pressure. Younger age, higher level of education and living partner or elderly was significantly (*p* < 0.05) associated with lower systolic and diastolic blood pressure. Overall, this study showed that knowledge toward healthy salt intake, BMI, education level, and living with others were significantly associated with the blood pressure among the elderly. Further education and intervention is required to improve knowledge on healthy salt intake among elderly as part of the prevention from hypertension.

## Introduction

A recent study by NCD Risk Factor Collaboration (2017) (1) revealed that Asian countries, especially industrial countries are facing a threat for a hypertension epidemic. The World Health Organization (WHO) predicted that 1.56 billion (29.2% of the world population) will develop hypertension by 2025. The prevalence of hypertension in Malaysia (i.e., 22.9%) is reported to be higher than its neighboring countries, i.e., Singapore (14.6%) and Thailand (22.3%) (2). Prevalence of hypertension increases with age and most are asymptomatic at least until the early stage. More than half of the population aged 65 years old and above in Malaysia has hypertension (3). Previous analysis based on the National Health Morbidity Survey (NHMS) 2018 by The Institute for Public Health (IKU) (4) showed that the prevalence of hypertension among pre-elderly in Malaysia was 32.7% with 77.3% pre-elderly reportedly had hypertension screening in the past 12 months (4). Despite the alarming prevalence, the awareness toward hypertension and factors related to it is still low (5).

Risk factors of hypertension included Body Mass Index (BMI), smoking, arterial stiffness and resistance, and high dietary salt or sodium intake (6). A significant contributor to the daily intake of sodium in Malaysia was found to be in sauces and cooked food (7, 8). Previous study by Suzana et al. reported poor hypertensive control among Malaysians with hypertension was related to high salt diet eating habits (9). The high sodium consumption among Malaysians were reported to be a result of poor knowledge and practice toward reducing salt intake (8). Although it is found that poor knowledge contributes to higher salt consumption, the reason behind such result is unknown. Another study by Apidechkul (10) toward elderly in the rural area of Thailand revealed that high salt intake was the factor causing hypertension. Excessive dietary sodium intake is associated with an increased risk of hypertension, which in turn may be a major risk factor of stroke, cardiovascular diseases (CVDs), and kidney diseases (11). Apart from that, an increase in body weight and blood level glucose, triglycerides, and albumin too enhanced the risk of high blood pressure in Malaysian elderly (12). Thus, effective strategies should be implemented to prevent further increase of hypertension. According to WHO (2), some population-level strategies that can be applied includes awareness campaigns on salt-reduction, education on food labeling and reformulation of processed foods.

Previous study by Norimah et al. (13) reported nutrition knowledge was poor among elderlies in Malaysia. Other prior studies also revealed that elderly with poor salt-related knowledge had poorer salt-related practices. Those with poor knowledge and lower risk perception regarding hypertension are the most potential to be exposed to this health problem and other health effects related to hypertension (14). A study in China also reported that elderly with poor knowledge and awareness toward hypertension contribute to the increase in hypertension cases in the country (15). Furthermore, poor awareness toward hypertension may increases the risk of the onset of other complications related to hypertension especially in older population (16).

Since hypertension is an arising global problem, collection of epidemiology data and assessing knowledge, attitude and practice related to healthy salt intake in a population is important in the construction of effective salt-reduction strategies (17, 18). To the best of our knowledge, there was no study that have reported on salt awareness among the elderly in Malaysia. Not only that, available researches on KAP does not state the reason behind such scores. Therefore, this study aimed to understand and assess the factors affecting the knowledge, attitude and practice on salt intake among the elderly in Bangi.

## Materials and Methods

### Study Design

This is a cross-sectional study aimed to determine the Knowledge, Attitude and Practice (KAP) on salt intake and its relationship with blood pressure among Malay elderly residing in a semi urban area of Klang Valley in Malaysia, i.e., Bandar Baru Bangi, Selangor, Malaysia. Sample size calculation was done using the Cochran (1977) (19) formula. The expected population proportion of 61.3% was taken from previous MySalt 2015 study (20).

(1) $$\begin{array}{l} n=\frac{{\left(Z_{\alpha /2}\right)}^{2}p\left(1-p\right)}{\Delta^{2}} \end{array}$$

Where by,

*n* = sample size 0.91134661

(Z_∞/2_) = confidence level of 95% (standard deviation 1.96)

P = population = 61.3% = 0.613 (expected proportion of population based on MySalt 2015 study)

Δ = accuracy level = 10% (0.10)

Therefore,

(2) $$\begin{array}{l} n=\frac{\left(1.96^{2}\right)\left(0.613\right)\left(1-0.613\right)}{0.1^{2}} \end{array}$$

= 91 subjects

Considering drop-out factors to be 10%, the real sample size is calculated as following:

Sample size (*n*) = 91 + (10% x 91)

= 100

A total of 94 elderly subjects comprising of 40 males and 54 females were recruited through convenience sampling. Convenience sampling is described as non-probability sampling in which people are sampled because they are the easiest to reach. In this study, sampling was carried out in mosques as the community there were the easiest to communicate and contact with. Prior day of data collection, posters promoting the study was disseminated around the mosque and via Whatsapp (done by Head of Mosque). Data collection day was scheduled on the same day as mosque's religion class as a way to gain more elderlies to be approached after. Subjects selected are Malaysian, aged 60–81 years old, able to understand Malay or English and residing in Bandar Baru Bangi. Ethics approval was obtained from Universiti Kebangsaan Malaysia (UKM) Research Ethics Committee (reference number: UKM.PPI.800-1/1/5/JEP-2019-525).

### Data collection

This study was initially planned to involve multi-ethic population whereby data will be collected at community centers in the area. However, due to time constraint (3 months for data collection), we were not able to obtain the permission from the respective Municipal Council on time (up to months for approval). We then decided to approach the board in churches, temples and mosques in the area to join the study, but only the Muslim or majority Malay community allowed us to collect data and were able to accommodate our needs (space to collect data, tables, and chairs) while others were either not interested or was not able to accommodate due to already scheduled events.

Thus, data were collected from a sample of 100 Bangi residents aged 60 and over at the time of the study. The study was conducted by interview administered questionnaire in Masjid Al-Umm and Masjid Al-Hasanah in Bandar Baru Bangi by six students from Nutrition background in July 2019 to October 2019. Subjects were invited to take part in the research via text blasts and mosque announcements. A few stations were set up for the collection of sociodemographic, anthropometric and KAP data. After excluding incomplete data, there were 94 eligible subjects to participate in the survey. Willingness to participate in the survey was taken to imply consent. Survey was conducted in Malay and English.

### Sociodemographic and Health Profiles

Subjects were required to fill in forms regarding sociodemographic information and health history at designated stations. Sociodemographic information includes gender, race, age category (60–69, 70–79, and over 80 years old), marital status, education level, living alone or with family or partner, smoking history, sleep schedule and alcohol intake. For health profiles, subjects were required to jot down related health history and medications taken for it. Only the most prominent health diseases were chosen i.e., Hypertension, Diabetes, and Hypercholestrolemia.

### Knowledge, Attitude and Practice (KAP) Questionnaire

This questionnaire was adapted from previous studies by Institute of Public Health (20) (Module C) and modified to meet objectives of the study. The Knowledge, Attitude and Practice (KAP) Questionnaire comprises of three modules: Module A, Module B, and Module C. Module A includes sociodemographic and personal information of subjects such as name, gender, race, and education level. Module B consists of subject's health history whereas Module C consists of 18 questions that assess knowledge, attitude, and practice of salt intake. Practice of salt intake was assessed using the adopted and modified MySalt 2015 Knowledge, Attitude and Practice (KAP) questionnaire (20). In this questionnaire, questions related to salt intake practices were included as the following: Do you add extra soy sauce or sauce into food when you eat? How much salt is used when preparing food at home? Do you request for less salt when eating out? How much salt do you think you take every day? Is controlling salt intake your daily routine? If yes, how do you control it?

Each answer in Module C was scored in order to classify subject based on scoring by Bakaman et al. (1996) (21) as follows:

**Table:** 

**Score**	**Knowledge**	**Attitude**	**Practice**	**Total KAP**
<60	Poor	Negative	Inadequate	Poor
60–70	Fair	Neutral	Adequate	Fair
>70	Good	Positive	Good	Good

Subjects who scored more than 70 for each part were classified as having a good and satisfying knowledge, attitude, and practice on salt intake. The questionnaire was validated through a pilot study among 80 subjects in Selangor and Kuala Lumpur. Findings from the pilot study were analyzed using Cronbach Alpha to determine the reliability of the questionnaire. A value of 0.7 Cronbach Alpha was obtained, proving that this questionnaire is indeed reliable (22). A dual language version of this questionnaire was also created to ensure subjects have a better understanding of each of the questions.

### Anthropometric Measurement

Anthropometric measurement of subject is conducted according to International Society for the Advancement of Kinanthropometry (ISAK) protocol (23). Measurements consist of height, weight, body fat percentage, hip and waist circumference and blood pressure. SECA Bodymeter 213 (SECA, Germany) was used to measure height with accuracy nearest to 0.1 cm. For weight, a SECA 880 Digital Weighing Scale was used with accuracy nearest to 0.1 kg. The instrument used to measure body fat percentage is Omron HBF-306 (hand-held BIA). Waist and hip circumference were measured using Lufkin tape with accuracy nearest to 0.1 cm whereas Omron HEM-7120 was used to measure blood pressure. Classification of blood pressure is adopted from Malaysia's Clinical Practice Guidelines: Management of Hypertension 2018 (24).

### Statistical Analysis

Statistical analysis was performed using IBM SPSS version 23.0. The significance level in this study was set to 0.05, any *P* < 0.05 was denoted as statistically significant. Descriptive analysis was used to determine sociodemographic characteristics of subjects. Data with normal distribution are tested with parametric test (Independent *T*-Test, Pearson's Chi-Square) whereas data that are not normally distributed are tested with non-parametric test (Spearman's Rho). Independent *T*-Test was used to determine on anthropometric and blood pressure measurements on male and female subjects. Pearson's Chi Square was used to determine the relationship between KAP scores on sociodemographic factors and blood pressure. Spearman's Rho was used on sociodemographic and anthropometric measurement of elderlies on blood pressure.

## Results

Table 1 shows the sociodemographic data of the subjects. Majority of the subject in this study were females (57.5%). This study involved only Malays as there were no response from Chinese or Indian community centers. More than half of the subjects in this study were aged 60–69 years old (88.3%) and were married (88.3%). Majority subjects were also reported to have tertiary education level (60.6%). Most subjects were reported to live with family or spouse (97%). As for lifestyle, a greater number of subject had no smoking history (98%), claims to be not under pressure or stress (93%), gets enough sleep (93%), and abstains alcohol (100%).

**Table 1:** Sociodemographic data of subjects.

**Characteristics**	**Total (*n* = 94)**	**Percentage (%)**
**Gender**		
Male	40	42.5
Female	54	57.5
**Age**		
60–69 years old	83	88.3
70–79 years old	10	10.6
80 years old and above	1	1.1
**Race**		
Malay	94	100
**Marital status**		
Married	83	88.3
Divorced	9	9.7
Widower/ Widow	2	2
**Education level**		
Secondary education	37	39.4
Tertiary education	57	60.6
**Residence**		
Alone	3	3
With spouse/family	91	97
**Smoking history**		
Yes	90	96
No	2	2
	92	98
**Adequate sleep (7-8 hours)**		
Yes	87	93
No	7	7
**Alcohol intake**		
Yes	0	0
No	94	100

Table 2 shows the anthropometry data of subjects according to gender. There was a significant difference (*p* < 0.05) in body weight, height, body fat percentage, and waist-hip ratio between male and female subjects. Men were found to be heavier, taller, and have bigger waist-hip ratio compared women and women have relatively higher body fat percentage than men. There was no significant difference (*p* > 0.05) in Body Mass Index (BMI), waist and hip circumference and systolic and diastolic blood pressure between genders. There was no not significant difference in terms of normal BMI between men and women. However, there more overweight (34%) women compared to men (11%) and vice versa for obesity where men (14%) were found to be more obese than women (6%).

**Table 2:** Anthropometry characteristics of subjects.

**Anthropometry characteristics**	**Men (*n* = 40)** **(Average± s.d.)**	**Women (*n* = 54)** **(Average± s.d.)**	**Total (*n* = 94)** **(Average± s.d.)**	***P*-value**
Weight (kg)	72.98 ± 16.15	60.93 ± 10.22	64.82 ± 13.38	0.001
Height (m)	162.46 ± 5.96	151.59 ± 5.19	155.37 ± 7.34	0.000
Body Mass Index (kg/m^2^)	27.31 ± 5.19	26.46 ± 4.16	26.71 ± 4.44	0.993
Percentage Body Fat (%)	26.96 ± 8.23	35.57 ± 6.11	32.76 ± 7.95	0.001
Waist circumference (cm)	94.77 ± 14.99	86.72 ± 10.09	89.52 ± 12.48	0.210
Hip circumference (cm)	102.87 ± 10.64	98.75 ± 8.05	100.13 ± 9.08	0.214
Waist-Hip Ratio (WHR)	0.92 ± 0.07	0.86 ± 0.06	0.88 ± 0.07	0.002
Systolic Blood Pressure (mmHg)	139.91 ± 17.03	131.28 ± 21.76	134.16 ± 20.11	0.235
Diastolic Blood Pressure (mmHg)	78.33 ± 10.76	73.11 ± 10.54	74.08 ± 10.83	0.520

*Independent T-Test was used to compare between the parameters between genders*.

Table 3 shows the score for each knowledge, attitude and practice (KAP) on salt intake among subjects. There was a slight difference in the number of subjects who scored fair (38%) and poor (39%) for knowledge on salt intake and the consequences of having too much salt. Majority subjects (75.5%) were reported to have positive attitude toward a healthier salt intake and believe that a lower salt intake will benefit their health in the long run. Majority of the subjects (57.4%) had average overall score of KAP. Although majority of the subjects had positive attitude toward healthy salt intake, they have poor to fair knowledge and poor practice on healthy salt intake.

**Table 3:** Scores of knowledge, attitude and practice on salt intake among subjects.

**Part**	**Marks**	**Average score (mean ± s.d.)**	**Total (*n* = 94)**	**Percentage (%)**
Knowledge	<60% (Poor)	49.97 ± 8.58	37	39
	60–70% (Fair)	64.52 ± 2.05	36	38
	>70% (Good)	73.47 ± 3.46	21	22
Attitude	<60% (Negative)	58 ± 0	3	3.2
	60–70% (Neutral)	62 ± 1.77	20	21.3
	>70% (Positive)	86.15 ± 8.65	71	75.5
Practice	<60% (Poor)	52.13 ± 6.01	46	48.9
	60–70% (Adequate)	62.28 ± 2.25	35	37.2
	>70% (Good)	73.77 ± 3.65	13	17.3
Overall score for knowledge, attitude and practice	<60% (Poor)	54.18 **±** 3.46	11	11.7
	60–70% (Average)	65.81 **±** 3.29	54	57.4
	>70% (Good)	74.55 **±** 3.28	29	30.9

The association between anthropometry measurements and KAP variables of elderly with blood pressure is shown in Table 4. Based on the findings, Body Mass Index (BMI) correlated significantly (*p* < 0.01) with systolic and diastolic blood pressure. For KAP scores, knowledge was significantly (*p* < 0.05) associated with systolic blood pressure.

**Table 4:** Association of anthropometric and KAP characteristics with blood pressure.

**Anthropometric characteristics**	**Systolic blood pressure (mmHg)**		**Diastolic blood pressure (mmHg)**	
	***r***	***p***	***r***	***p***
Body mass Index (BMI)	0.278	0.000^**^	0.202	0.001^**^
Weight (kg)	0.038	0.769	−0.028	0.838
Height (cm)	0.025	0.849	−0.022	0.865
*Waist-Hip Ratio*	0.205	0.12	0.051	0.699
Waist Circumference (cm)	0.066	0.608	0.115	0.371
Hip circumference (cm)	0.102	0.424	0.065	0.61
**KAP scores**	**Systolic blood pressure (mmHg)**		**Diastolic blood pressure (mmHg)**	
	***p***		***p***	
Knowledge	0.037^*^		0.094	
Attitude	0.28		0.936	
Practice	0.457		0.234	
Overall KAP	0.069		0.544	

*Spearman's Rho was used to test anthropometry data against blood pressure*.

* *Correlation is significant at p < 0.05*.

** *Correlation is significant at p < 0.01*.

Chi-Square was used to test KAP scores against blood pressure. ^*^p-value is significant at p < 0.05.

Table 5 shows age was significantly (*p* < 0.05) correlated with the attitude score while education level was significantly (*p* < 0.05) correlated with the knowledge score. An inverse significant association (*p* < 0.01) was observed between education level and blood pressure where elderly with higher education level (university level) had a lower and more controlled systolic and diastolic blood pressure.

**Table 5:** Association of knowledge, attitude and practice scores on sociodemographic characteristics.

**Sociodemographic characteristics**	**Knowledge score (K)**	**Attitude score (A)**	**Practice score (P)**	**Overall KAP score**
	***p***	***p***	***p***	***p***
Gender	0.084	0.111	0.951	0.637
Age	0.201	0.006^*^	0.795	0.169
Marital Status	0.986	0.12	0.276	0.583
Education level	0.020^*^	0.313	0.738	0.826
Living alone/ with company	0.898	0.203	0.211	0.813

*Chi-Square test was used*.

* *p-value is significant at p < 0.05*.

## Discussion

In this study, knowledge, attitude and practice (KAP) toward salt intake among Malay elderly residing in Bandar Baru Bangi and its association with blood pressure were assessed. Based on the data obtained, this study showed that the knowledge on salt among the majority of elderly in Bangi ranged from poor to moderate. Further investigation in this study found that although elderly had knowledge about the relationship of salt and hypertension, many was not aware of other diseases such as osteoporosis, kidney stones, and gastric cancer that may also develop with high dietary salt intake. The findings of this study are in accordance with previous study in China by Song et al. (10) who reported a poor knowledge on salt-related disease among the elderly, that needs to be improved. However, a study by Grimes et al. (25) revealed the opposite in which elderly in Australia were reported to be more likely aware of the link between excessive salt intakes with certain specific health conditions.

Apart from that, majority of the elderly in this study have no knowledge on the daily salt intake recommendation set by the Ministry of Health Malaysia as stated in the Recommended Nutrient Intake (RNI) Malaysia, which is 5 g/daily. Therefore, this study deduces that elderly have lack of specific knowledge on the maximum daily dietary salt intake. This may be due to health education regarding salt intake and hypertension and salt-related education are only taught on the surface (26). Based on the interview conducted in this study, elderly only had access to nutrition and health knowledge when they are referred to nutritionists or dietitians in the hospital. In Hong Kong, elderly living in Elderly Health Centers (EHC) were reported to have better health literacy than the general elderly population as they had access to primary health care (27). Another study by Lee et al. (28) suggested that minority of the population in the Republic of China had worse nutritional status and poorer health as they often had limited access to health care information and centers. Thus, it is observed that nutrition and health knowledge is only accessible for the elderly if they had access to health care. This shows that there is a need to ameliorate the education and health campaigns to further improves the understanding and knowledge of the elderly especially on other salt-related diseases and salt-reduction knowledge.

In terms of attitude, majority of elderly in this study had positive attitude toward salt reduction and healthy salt intake. This study found that elderly showed effort in reducing salt intake and acknowledge the importance of consuming salt according to the recommendation. This finding is concurrent with previous studies by Zhang et al. (26) and Lee et al. (28) where elderly was reported to believe that salt reduction would indeed give positive impact to health status. However, it is important to note that a small number of the subjects in this study who were older had negative attitude toward healthy salt intake as they are used to eating salty food and believe that salt has no correlation to the onset of poor health conditions.

This study found that almost half of the study population (48.9%) had poor practice scores. It was found that majority of them do not request for salt to be reduced in the cooking when eating out as they believe it is beyond their control to request for such. Furthermore, the elderly did not read salt content on the labels when making choices during grocery shopping, as their main concern was more on the price and its halal status. In addition, some elderly in this study also did not find the need to control their salt intake as they believed that the amount of salt consumed daily is just right. This finding is similar to a study by Cheikh et al. (29) that reported a low percentage in salt intake control was due to the believe that their salt intake is within the right amount.

Although elderly had a positive attitude toward healthy salt intake, the salt-related knowledge and practice were inadequate and unsatisfying. Previous studies reported the same findings where positive attitude toward salt intake does not translate to good salt control practices (30, 31). Thus, awareness campaigns and salt-control interventions in the community need to be revised for improvements with emphasis on the elderly. One way is by improving knowledge on salt-reduction and its benefits as it will increase effectiveness of salt-reduction initiatives (26). Having knowledge will influence one's behavior as behavior is determined by intention and intention is influenced by how one perceives knowledge (32). Therefore, if elderly had better knowledge on the health complications and problems related to excessive salt intake, they might be more prone to reduce dietary salt intake, thus improving their salt-related practices.

This study revealed that elderly with higher BMI regardless of gender had higher systolic and diastolic blood pressure. This finding is parallel to prior studies where mean systolic and diastolic blood pressure was higher among older subjects with elevated BMI (33–35). Based on this finding, the study can conclude that hypertension can be controlled and prevented by reducing BMI. Apart from that, knowledge and attitude scores were also to have significant relationship with certain sociodemographic variables. Subjects that obtained tertiary education such as Degree or Diploma were reported to have higher knowledge scores compared to those who obtained secondary education. Elderly aged 60–70 years old were observed to have a more positive attitude toward healthy salt intake.

This study showed that subjects with higher knowledge scores had better systolic blood pressure. Only the knowledge section of the KAP questionnaire was found to be significantly associated with lower systolic blood pressure. It can be concluded that those with better knowledge and awareness on the risk factors and related complications and of hypertension had better control of their blood pressure. This finding is comparable to findings by Zhang et al. (26) that reported that subjects that had more knowledge on risk factors and hypertension complications as well as higher awareness had better blood pressure control. Therefore, it is clear that by improving the knowledge of elderly on healthy salt intake will have better association with their blood pressure reading. The overall scores of KAP questionnaire for the elderlies were non-satisfactory. Score classifications were referred from the Journal of Family and Community Medicine (21). However, it was found that their attitudes toward healthy salt intake were mainly positive.

The limitation of this study is the unavailable data on salt intake level from diet of the studied population. Due to small sample size, a robust statistical analysis to identify associated factors of KAP level could not be done. There may also be bias to the KAP questionnaire as practices reported may not be subject's daily practice. The strength of the study is, it was conducted among a unique population that might provide a different perspective for intervention in future studies. This study is the first in Malaysia that explores the KAP of elderlies on salt intake.

## Conclusion

Salt-related knowledge and practice among elderly in this study was unsatisfying despite having positive attitudes toward healthy salt intake. Higher education level was significantly associated with higher knowledge score whereas a younger age of 60–69 years old was significantly associated with higher attitude scores. Higher knowledge scores were also observed to significantly associated with controlled systolic blood pressure. Older age and living alone was significantly associated with higher blood pressure whereas higher education level was significantly associated with lower blood pressure. add limitation if this study, strength, and implication. Future study should be looking into the salt intake of the elderly through diet food record. Further education and intervention is required to improve knowledge on healthy salt intake among elderly as part of the prevention from hypertension.

## Data Availability Statement

The original contributions presented in the study are included in the article/supplementary files, further inquiries can be directed to the corresponding author.

## Ethics Statement

The studies involving human participants were reviewed and approved by National University of Malaysia. The patients/participants provided their written informed consent to participate in this study.

## Author Contributions

HH, HY, and SS contributed conception and design of the study. NK organized the database and performed the statistical analysis. NK and HH involved in preparing the first draft of the manuscript. All authors contributed to manuscript revision, read, and approved the submitted version.

## Conflict of Interest

The authors declare that the research was conducted in the absence of any commercial or financial relationships that could be construed as a potential conflict of interest.
